# Genetic insights into canine flank alopecia in Rhodesian ridgebacks: identifying candidate genes

**DOI:** 10.1016/j.vas.2026.100670

**Published:** 2026-04-21

**Authors:** Millie U.M.Y. Verschuuren-Tjoeng, Lieke Vree, Claudia Rozendom, Yvette Schlotter, Ronette Gehring, Frank G. van Steenbeek

**Affiliations:** a Utrecht University, Faculty of Veterinary Medicine, Department of Clinical Sciences, Yalelaan 104 3584 CM Utrecht, The Netherlands; b Veterinaire Dermatologie Consultancy, Culemborg, The Netherlands; c Utrecht University, Faculty of Veterinary Medicine, Department of Population Health Sciences, Yalelaan 104 3584 CM Utrecht, The Netherlands

**Keywords:** Genome-wide association, Low-pass sequencing, *SPTB*, *SYNE2*, *EPB41L1*, *DPY19L1*, *MAPK4*

## Abstract

Canine flank alopecia (CFA) is a recurrent, seasonal, non-inflammatory skin disorder of uncertain etiology that affects several breeds, including Rhodesian ridgebacks. While endocrine and environmental influences have been suggested, the genetic background remains unclear. This study aimed to identify candidate genes associated with CFA in Rhodesian ridgebacks using a genome-wide approach.

Low-pass whole-genome sequencing with imputation was performed on 48 Rhodesian ridgebacks (24 cases, 24 controls), followed by genome-wide association analyses and evaluation of protein-coding variants. Filtering for variants with predicted functional impact revealed variants in *SPTB, SYNE2*, EPB41L1, and DPY19L1, which are involved in cytoskeletal function, cell adhesion, neural signaling, and pathways critical for hair follicle cycling, such as Wnt, BMP, MAPK, Notch, and Hedgehog signaling.

Our findings demonstrate that CFA is likely a skin disorder with genetic heterogeneity, where multiple loci and possibly modifier variants influence disease susceptibility, rather than a simple monogenic disorder. These results align with the complex inheritance observed in atypical recurrent flank alopecia, supporting a complex multifactorial etiology involving both genetic and environmental factors.

This study provides the first insights into the genetic background of CFA in Rhodesian ridgebacks and highlights candidate genes for further functional investigation. Broader genomic approaches are needed to elucidate the disorder’s genetic basis, ultimately guiding CFA-preventative breeding strategies.

## Highlights

• CFA is a skin disorder that is unlikely to be caused by a single genetic defect.
• A potential genomic region may warrant further investigation in relation to CFA.
• Preliminary genomic insights into CFA support future larger-scale investigations.
• Growing understanding of the genetic and biological mechanisms underlying CFA.

## Introduction

Hair (haircoat) plays a vital role in both humans and other mammals. In most mammals, it provides thermoregulation (insulation against cold climate conditions), protection against external influences (e.g., sun exposure and mechanical injuries), and plays a role in social communication (e.g., facial whiskers in rodents and cats serve as tactile sensors). The hair follicle (HF) is a sac-like structure in the skin from which the hair grows (Lin et al., 2022; Park et al., 2018). The HF goes through several hair growth cycles (HC) throughout life: growth (anagen), regression (catagen), rest (telogen), and shedding (exogen) (Schmidt-Ullrich & Paus, 2005; Welle & Wiener, 2016). Disruption of these growth phases can lead to changes in hair quality or quantity, potentially resulting in non-inflammatory alopecia (Rishikaysh et al., 2014; Stenn & Paus, 2001).

**Fig. 1:**
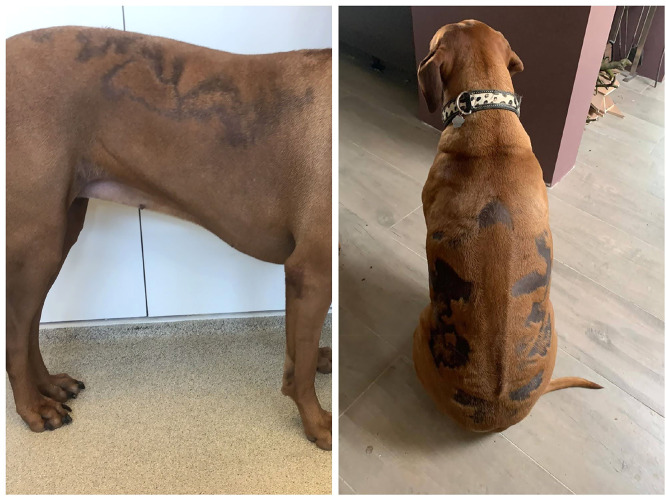
Phenotypic presentation of CFA in Rhodesian ridgebacks. Bilateral, sharply marginated alopecia in the thoracolumbar region with lesional hyperpigmentation.

Canine flank alopecia (CFA) is a skin disorder in dogs characterized by seasonally recurring episodes of non-inflammatory, non-itchy, often hyperpigmented, well-defined patches of symmetrical hair loss, typically located in the thoracolumbar region (Fig. 1).

Over the years, several terms have been used to describe CFA, including seasonal flank alopecia, idiopathic cyclic flank alopecia, cyclic follicular dysplasia, and recurrent flank alopecia (Daminet & Paradis, 2000; Muntener et al., 2012). Typically, hair loss begins between November and April, during months with shorter daylight hours (Paradis, 2009). The hair loss is generally bilateral but may occasionally be confined to one side of the body.

Additionally, an atypical form of canine recurrent flank alopecia (aRFA) has been described anecdotally in certain breeds, including the Cane Corso and the Cesky Fousek. Dogs affected with aRFA display not only recurrent alopecia on the flanks, but also on the sacral area, the thighs, the base of the tail, and sometimes the bridge of the nose and the ears (Neradilova et al., 2022; Vandenabeele, Declercq, et al., 2013).

CFA is commonly seen in Rhodesian ridgebacks and predominantly affects young adult individuals. The mean age of onset of the first episode is approximately 4 years (range: 1-11 years), although most cases develop CFA for the first time between 3 and 6 years old. Both male and female dogs can be affected, regardless of their reproductive status (Miller et al., 2012; Paradis, 2014; Verschuuren et al., 2024). In CFA-affected dogs, hair usually regrows spontaneously within 3-8 months. The new hair is generally normal in quality and density, although some dogs may regrow darker hair in the affected areas. Regrowth can be complete or partial before the next hair loss cycle occurs. Affected dogs are otherwise healthy, and the condition poses mainly a cosmetic concern. However, the affected hairless skin is more susceptible to sunburn and bacterial or fungal skin infections (Welle, 2023). Additionally, the characteristic recurrent pattern often causes frustration for both breeders and dog owners. Despite its prevalence, little is known about the pathogenesis and genetic basis of CFA.

The seasonal nature of CFA suggests an association with photoperiod, implying that the light-dependent neurohormone melatonin may play a role in the pathogenesis of CFA (S. I. Vandenabeele et al., 2014). Melatonin is known to affect hair growth physiology (Fischer et al., 2008; Liu et al., 2022; Rose et al., 1984). The potential role of melatonin in hair growth response has been studied in dogs (Verschuuren et al., 2022) and other species, such as sheep and mouflon (Lincoln & Richardson, 1998; Santiago-Moreno et al., 2004).

Furthermore, a genetic basis of the condition is suspected because certain breeds, such as Boxers, Bulldogs, Airedale terriers, and Rhodesian ridgebacks, are more prone to developing CFA (Miller & Dunstan, 1993; Muntener et al., 2012). However, CFA may also be influenced by environmental, epigenetic factors, and multiple genetic factors (Paradis, 2012; Vandenabeele, DeCock, et al., 2013). Therefore, in the pathogenesis of CFA in Rhodesian ridgebacks, various genes may be involved, as reported in the Cesky Fousek (Neradilova et al., 2022).

Technological advances in genetic research offer new opportunities to gain a deeper understanding of the genetic background of various hereditary skin disorders in dogs. Next-generation sequencing (NGS) technologies, especially low-pass whole-genome sequencing, have emerged recently as a cost-effective, powerful technique to pinpoint genetic variants associated with diseases and phenotypic traits. By sequencing the genome at lower coverage and imputing missing data, genetic variants can be mapped with greater efficiency (Bell et al., 2023; Bougiouri et al., 2025).

Our previous study combined *melanophylin* genotyping and pedigree analysis, hypothesizing an autosomal, possibly monogenic background (Verschuuren et al., 2024). We found that CFA in Rhodesian ridgebacks is unlikely to be associated with mutations in the *MLPH* gene. However, findings from the pedigree analysis suggested the implication of a genetic component in the pathogenesis of CFA. In the current study, we focus on identifying genetic variants associated with the occurrence of CFA in Rhodesian ridgeback dogs using low-pass sequencing and conduct a genome-wide association study (GWAS). Understanding the genetic basis of this condition could offer valuable insights into its underlying pathophysiology, ultimately leading to better diagnostic tools and breeding strategies.

## Materials and methods

### Sample collection

Control DNA samples were obtained from the DNA databank of the Expertise Centre for Veterinary Genetics (ECVG), Utrecht University, the Netherlands. For this study, DNA samples from Rhodesian ridgebacks with no history or clinical signs of CFA were used. Control dogs were selected to represent the same breed population as the affected dogs. Sex matching between affected and control dogs was not considered essential for the study design, as CFA is reported to occur in both sexes of all reproductive statuses, and is not considered a sex-linked condition (S. Vandenabeele et al., 2014). Samples were collected as EDTA blood samples during routine veterinary visits. All samples in the ECVG databank are stored and used for research purposes with informed owner consent.

DNA of affected dogs was isolated from surplus blood samples collected in two previous studies used for hematological and serum biochemical investigation (Verschuuren et al., 2024; Verschuuren et al., 2022). The study population in those studies consisted of privately owned Rhodesian ridgebacks in the Netherlands. We obtained written consent for the secondary use of the samples and ensured that the data were pseudonymized.

In the 2022 study (Verschuuren et al., 2022), the inclusion criteria were dogs that had suffered at least two consecutive CFA episodes, with onset between late fall and spring, followed by spontaneous hair regrowth in summer. Dogs that had received any CFA treatment were excluded from that study to ensure accurate clinical history and diagnosis of CFA. Included dogs underwent general physical and dermatological examinations, and blood sampling. If the test results showed no abnormalities, two 6mm skin biopsies were subsequently collected from affected alopecic skin to confirm that pathohistological findings were consistent with CFA.

In the 2024 study (Verschuuren et al., 2024), eligibility was assessed by requesting owners to complete a questionnaire, submit photographs of their dog exhibiting active CFA, and provide pedigree information. Upon inclusion, the attending veterinarian collected EDTA-treated blood samples for hematological and routine biochemical testing, including total-T4 and cortisol serum concentration, to rule out other causes for noninflammatory alopecia, especially hypothyroidism and hypercortisolism. As CFA is primarily a clinical diagnosis based on characteristic lesion distribution, seasonal recurrence, and the exclusion of endocrinopathies or other underlying disorders, histopathological examination was not routinely performed for dogs included in the 2024 study. For the current study, the surplus whole blood in EDTA was sent to a specialized laboratory for DNA extraction. DNA was isolated using a MagCore® automated HF16 plus nucleic acid extractor (RBC Bioscience).

## Genetic analysis

### Low-pass WGS

A set of 48 Rhodesian ridgebacks (24 cases and 24 controls) was genotyped by low-pass sequencing with imputation using skimSEEK™ technology (Neogen Europe Ltd). In short, sequencing libraries were prepared from DNA using the Kapa HyperPlus Library Preparation Kit by Illumina, and low-pass whole-genome sequencing was performed on an Illumina NovaSeq 6000 instrument, generating dual-index 150 bp paired-end reads. Raw reads were mapped against CanFam3.1. Imputation of genotypes from sequencing data was performed using loimpute v. 0.18 by Gencove, Inc. (New York, NY) to a reference panel comprising high coverage WGS data of 676 dogs covering 91 dog breeds, of which four were Rhodesian ridgebacks.

## GWAS

Population structure was assessed to evaluate potential stratification within the study cohort. Multidimensional scaling (MDS) analysis was performed based on genetic similarity between individuals, as implemented in the GenABEL package in R (Aulchenko et al., 2007). We analyzed the data by performing a genome-wide association study (GWAS) and by whole-genome sequence variant analysis.

The GWAS was performed using GEMMA v0.98 (Zhou & Stephens, 2012) to identify genetic variants associated with CFA. A linear mixed model was fitted to account for population structure and relatedness among individuals by incorporating a genetic relationship matrix (GRM) as a random effect. The GRM was estimated from high-quality SNP genotypes after standard quality control filtering (minor allele frequency > 0.05, missingness < 0.05, and Hardy–Weinberg equilibrium p > 1 × 10⁻⁶). Linkage disequilibrium (LD) pruning was performed to obtain a set of approximately independent SNPs by removing correlated variants based on a pairwise LD threshold of r^2^<0.8.

Association testing under the same QC thresholds was conducted using the Likelihood Ratio Test implemented in GEMMA, which compares the likelihoods of models with and without the SNP effect. This approach provides robust statistical inference while accounting for sample structure and relatedness. To assess the distribution of association test statistics and detect potential inflation due to population stratification or other confounding factors, a quantile-quantile (QQ) plot was generated comparing the observed versus expected -log10(P) values from the genome-wide association analysis. The expected distribution of P-values under the null hypothesis was derived from the uniform distribution. The genomic inflation factor (λ) was calculated as the median of the observed chi-square test statistics divided by the expected median of the chi-square distribution with one degree of freedom. The Manhattan plot and QQ plot were generated using the R package qqman.

### Variant analysis

For the variant analysis in the low-pass WGS data, DNA variants were filtered for their effect on protein by selecting variants with a HIGH or MODERATE effect defined by SnpEff (Cingolani, Platts, et al., 2012). SnpSift (Cingolani, Patel, et al., 2012) was used to perform a Fisher's Exact test per variant under an allelic model. The allele frequencies of the variants in the cohort were compared with those in the genomes provided by the Dog10K consortium (Meadows et al., 2023). Variants were cross-referenced against 1) a list of candidate genes (supplementary Table S1), which were selected based on their known involvement in mammalian HF development, structure, and function, as well as HC biology, along with the various involved molecular signaling pathways that highly regulate these biological processes (Bellani et al., 2025; Lee & Tumbar, 2012; Lin et al., 2022; Rishikaysh et al., 2014; Stenn & Paus, 2001), and 2) a list of genes differentially expressed in skin biopsies of dogs with atypical recurrent flank alopecia (Neradilova et al., 2022)(supplementary Table S2).

## Results

### GWAS

**Fig. 2:**
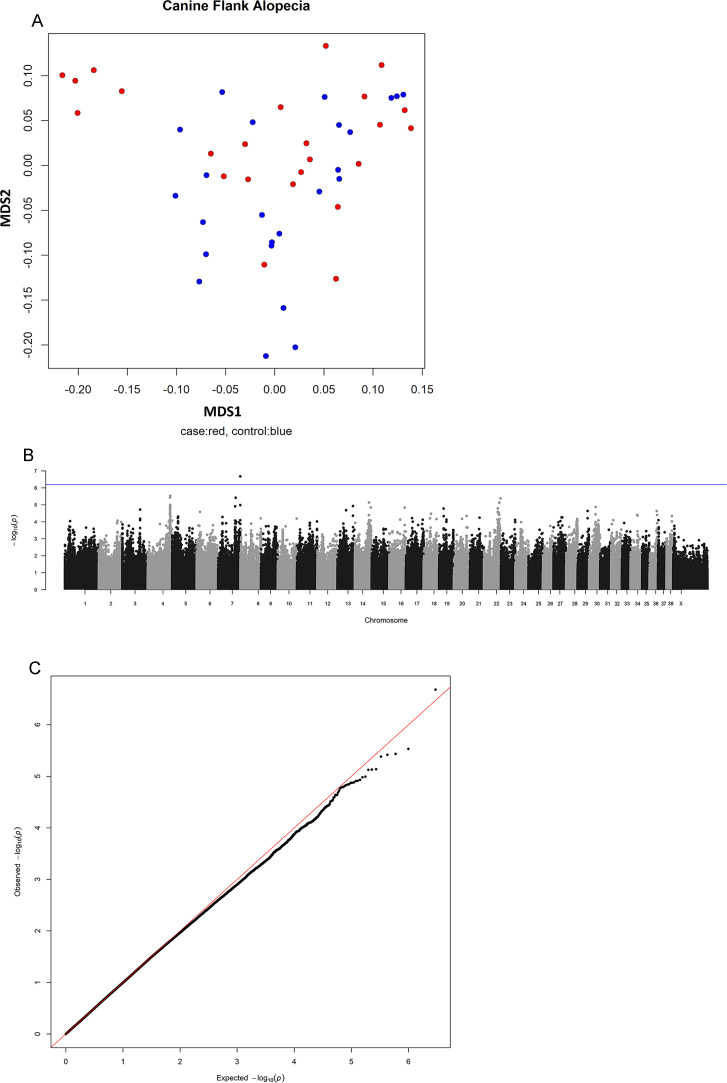
Results of the Genome-wide association scan of canine flank alopecia in Rhodesian ridgebacks. A) A multi-dimensional scaling plot depicting the structure of the study population with cases depicted in red and controls in blue. B) A Manhattan-plot of the Likelihood Ratio Test corrected for population structure showing a suggestive signal on chromosome 7. The blue line depicts the suggestive P-value calculated by 1/N(SNPs). C) Quantile–quantile (QQ) plot of observed versus expected −log10(p-values) from the GWAS, used to assess deviation from the null distribution and potential genomic inflation.

The average number of reads per sample was 10,731,174±1,278,127, corresponding to an average coverage of 0.65±0.08X. Per sample details are shown in Supplementary Table S3. An MDS-plot (Fig. 2A) did not reveal strong stratification of cases and controls with a corresponding genomic inflation factor (λ=0.99). After quality control and LD pruning, a total of 1,487,709 SNPs remained for association analysis. This analysis did not result in significant regions of interest after Bonferroni correction for multiple testing (Fig. 2B); however, suggestive signals were detected on chromosome 7. A quantile-quantile (QQ) plot was generated to assess the distribution of observed p-values against those expected under the null hypothesis, confirming the correction for potential inflation due to population stratification or other confounding factors (Fig. 2C). A zoomed regional association plot, along with the corresponding gene annotations, is provided in Supplementary Figure S1.

### Variant analysis

As the GWAS did not identify any genome-wide significant regions, making a monogenic basis unlikely, we further explored the dataset by focusing on coding variants predicted to affect protein function. Selecting the coding variants with a MODERATE and HIGH impact resulted in a total of 54,181 variants. 16 variants were associated with a p-value<0.0005, of which 11 showed a higher allele frequency of the alternate allele in the cases compared to controls (Table 1). All 11 selected variants with elevated allele frequencies showing low allele frequencies in the control group correspondingly exhibited similarly low frequencies in Dog10K.

**Table 1:** The most significant associated protein-coding DNA variants with a moderate or high effect.

CFA	POS	REF	VAR	effect	AF cases	AF controls	p-value	AF Dog10k
8	39187375	C	T	SPTB_p.Arg1114Gln	0.48	0.10	4.56E-05	0.17
11	46789067	A	T	ENSCAFG00000047734_p.Ser42Thr	0.31	0.02	8.26E-05	0.06
30	28642398	C	T	SNX1_p.Pro519Ser	0.23	0.63	8.44E-05	0.30
8	46643742	A	ACAAAAGCAACATAGAAGTCTTTACAGACCCAGATTCACC	ENSCAFG00000041661_p.Thr66_Thr67insLysAlaThrTerLysSerLeuGlnThrGlnIleHisPro	0.52	0.15	8.95E-05	0.10
22	50364171	T	C	MDH2_p.His23Arg	0.48	0.85	8.95E-05	0.31
22	50364210	G	A	MDH2_p.Thr10Met	0.48	0.85	8.95E-05	0.30
22	50364227	G	T	MDH2_p.Cys4*	0.48	0.85	8.95E-05	0.30
2	46761768	C	A	ENSCAFG00000045085_p.Trp136Leu	0.29	0.02	1.85E-04	0.13
24	24993969	C	T	EPB41L1_p.Pro131Leu	0.29	0.02	1.85E-04	0.02
27	33416661	A	ACAG	GPRC5D_p.Leu297_Pro298insGln	0.31	0.69	2.29E-04	NA
8	38419531	C	T	SYNE2_p.Ala369Val	0.46	0.13	3.02E-04	0.12
8	38454351	G	A	SYNE2_p.Gly1242Asp	0.46	0.13	3.02E-04	0.14
14	46753745	T	TGCTTGCTCCGCGCCTGCAGGACCATCTTGGCATAGTCGCGCCCGCTC	DPY19L1_p.His1fs	0.46	0.13	3.02E-04	0.001
14	46753595	C	T	DPY19L1_p.Arg51Lys	0.48	0.15	3.98E-04	0.15
17	63353862	G	A	ENSCAFG00000029051_p.Leu195Phe	0.48	0.15	3.98E-04	0.23
8	39006331	TGA	T	PPP1R36_p.Arg396fs	0.60	0.25	4.29E-04	0.29

Abbreviations: CFA, canine autosomal chromosome; POS, position; REF, reference; VAR, variant; AF, allele frequency.

None of these genes overlapped with the initial GWAS findings, the defined list of candidate genes, nor were they found to be differentially expressed in affected tissue in the Cesky Fousek (Neradilova et al., 2022).

## Discussion

This study represents a genetic study of CFA in Rhodesian ridgebacks, utilizing low-pass sequencing as an effective approach for variant discovery and genotyping. The analysis identified a single genomic region reaching suggestive significance, while no regions surpassed the genome-wide significance threshold. These findings indicate that the associated variants should not be interpreted as causal, and the biological relevance of nearby genes remains to be determined. Importantly, the absence of significant associations argues against a simple monogenic basis for CFA. Instead, the observed genetic signal and variability in clinical presentation support a model of genetic heterogeneity, in which multiple loci and potential modifier variants contribute to disease susceptibility. This complexity helps explain the variable expression of alopecia among dogs, even within the same breed. Clinically, these results highlight the limitations of relying on single-gene testing and emphasize the need for broader genomic approaches to better understand risk and guide management.

The first genetic study of recurrent CFA was conducted in the Cesky Fousek (Neradilova et al., 2022), focusing on the atypical recurrent flank alopecia (aRFA) phenotype. Although this phenotype is not entirely comparable to the more typical forms of CFA seen in the Rhodesian ridgeback, it is noteworthy that association signals were detected in close proximity to each other. The most significant DNA variants in the allelic model of SnpSift are flanking one of the associated loci detected in the Cesky Fousek (top SNP located at CFA8:43,341,287). While this overlap does not imply shared causality, it suggests a potentially meaningful genetic basis despite the clinical differences.

In CFA, recurrent episodes of often reversible hair loss are caused by disruption of the normal biology and cycling of the HFs. HFs contain large populations of stem cells (HFSC) that contribute to hair growth and regeneration (Lin et al., 2022). HF development, HFSC activity, and HC are regulated by a complex interplay between various transcription factors and molecular signaling pathways (Bellani et al., 2025). Key signaling pathways include Wingless-related (Wnt), Bone morphogenetic protein (BMP), Sonic Hedgehog (Shh), Notch, and Mitogen-activated protein kinase (MAPK) (Akilli Ozturk et al., 2015; Bellani et al., 2025; Lee & Tumbar, 2012). The Wnt- and Shh-pathways play crucial roles in HF biology through anagen induction, promotion, and differentiation (Kretzschmar & Clevers, 2017; Lee & Tumbar, 2012; Rompolas & Greco, 2014). In contrast, BMP signaling inhibits HF anagen induction by suppressing the activity of HFSCs (Zhang et al., 2006). Notch signaling promotes differentiation and maintenance of HF (Lee & Tumbar, 2012; Powell et al., 1998). The MAPK pathway is not only involved in HF development but also influences HFSC proliferation and differentiation (Guo et al., 2017). A recent study on the interaction between melatonin and the MAPK pathway in cashmere goats indicated that the *MAPK3* gene is involved in secondary HF development (Diao et al., 2023). Additionally, maternal melatonin in pregnant rabbits was found to contribute to HF development in their offspring by activating the MAPK pathway (Feng et al., 2025).

These pathways mentioned above play established roles in HF development, cycling, and regeneration. Future research is needed to explore their potential association with the pathogenesis of CFA.

Given the absence of a clear monogenic signal in the GWAS, we further explored the dataset described in Table 1 by prioritizing coding variants with potential functional relevance to the disease. The candidate genes discussed below were selected based on predicted impact and previously reported biological functions relevant to skin integrity and HF structure. However, any potential contribution of these genes to the pathogenesis of CFA, including through subtle or polygenic effects, remains speculative and should be interpreted with caution.

### Potential role of the *SPTB* gene in skin and metabolism

The *SPTB* gene (*Spectrin Beta, erythrocytic*), located on chromosome 8, encodes spectrin-beta protein and is predominantly expressed in erythrocytes, not in skin. Spectrin proteins are vital for maintaining the shape and flexibility of the erythrocyte cell membrane. In contrast, the *SPTBN1* gene (*Spectrin Beta, Non-Erythrocytic 1*), an important *SPTB* paralog, is highly expressed in various tissue types, including skin (Jung & Wu, 2024), particularly in fibroblasts (Jung & Wu, 2023).

Large-scale gene-phenotype analyses in transgenic mice identify *SPTBN1* among genes associated with “abnormal hair growth” (Harmonizome, 2024). While this association does not confirm a direct functional role, it may indicate that *beta spectrin* is involved in the regulation of hair growth. Several studies have demonstrated that specific spectrin types are expressed in keratinocytes of the human skin (Shimizu et al., 1995; Tuominen et al., 1996).

Additionally, *SPTB*-related pathways include the MAPK-family signaling cascades (Wu & Haw, 2017). MAPK pathways are crucial for HF development, proliferation, and differentiation, and they are involved in regulating HFSC activation (Guo et al., 2017). Notably, the *MAPK3* gene has been implicated in secondary HF development in cashmere goats (Diao et al., 2023), supporting a broader role for MAPK family members in HF regulation. Therefore, the identified *MAPK4* gene, located within the genomic region on chromosome 7, showing suggestive association signals, represents an interesting candidate gene for further investigation.

Currently, published studies that confirm the direct functional involvement of the *SPTB* gene in mammalian HF development are lacking. However, indirect evidence supports its potential role in hair growth regulation, warranting further research to explore the possible implications of the *SPTB* gene in the pathogenesis of CFA.

### Potential role of the *SYNE2* gene in skin and HF metabolism

The *SYNE2* gene, also known as *Nesprin-2* (spectrin repeat containing nuclear envelope protein 2), located on chromosome 8, encodes the Nesprin-2 protein. *SYNE2* is expressed in mammalian skin cells, including epidermal keratinocytes (Luke et al., 2008). Since HFs contain keratinocyte-derived cells, an association of *SYNE2* with HF metabolism is plausible.

Currently, data establishing a direct association between *SYNE2* and HF biology or hair growth are lacking. However, Nesprin-2 is proposed as a positive regulator of the Wnt-signaling pathway, which plays a crucial role in HF development and hair growth (Neumann et al., 2010). Impaired Wnt-signaling may negatively affect HF regeneration and maintenance either directly or indirectly through crosstalk with SHH-signaling (another key signaling pathway in HF biology). This may indicate an indirect supportive role for *SYNE2*/Nesprin-2 in HF biology. Future research is needed to investigate this potential role.

### Potential role of *EPB41L1* and *DPY19L1* in dermatomal neural modulation of the skin

The *EPB41L1* gene (Erythrocyte membrane protein band 4.1-like 1), located on chromosome 24, encodes protein 4.1N, which serves as a cytoskeletal adaptor in both neural signaling and cellular homeostasis (Yang et al., 2021). The cytoskeleton serves as a cell’s internal scaffold of protein filaments, providing its structural support and shape, and enabling internal transport of organelles and other structures through the cytoplasm (Cooper & Adams, 2000). *EPB41L1* is mainly expressed in neural tissues but also moderately expressed in epidermal keratinocytes and dermal fibroblasts in the skin. (*Human Protein Atlas proteinatlas.org*).

The *DPY19L1* gene (dpy-19 like C-mannosyltransferase 1), located on chromosome 14, in which an in-frame insertion of 39bp was found, is primarily known for its involvement in neural development. Studies demonstrating a functional association between *DPY19L1* and HF biology are lacking. However, a study on the transcriptional organization of adult murine epidermis identified *DPY19L1* gene expression in specific single-cell clusters of HFs, indicating its expression in HFSC-related cells in mice (Joost et al., 2016). A functional association between *DPY19L1* and HFSC biology remains to be established.

Notably, both *EPB41L1* and *DPY19L1* play critical roles in neural tissues.

The body’s skin surface is divided into a map-like pattern of dermatomes. A dermatome is an area of the skin that is innervated by sensory nerve fibers from a specific single spinal nerve root. Each dermatome corresponds to a particular segmental sensory innervation area of the skin, categorized into cervical, thoracic, lumbar, sacral, or coccygeal regions. However, the molecular pathways through which these nerves influence HF biology remain unknown.

Interestingly, in cases of CFA, seasonally recurring hair loss typically develops in the thoracic and lumbar regions, which align with specific dermatomes. This distribution pattern could imply that regional nerve input plays a role, and both *EPB41L1* and *DPY19L1* may be involved in the molecular pathways affecting HF biology, thereby linking dermatomal innervation to HF function. This assumption is supported by studies demonstrating that neural innervation of the skin is essential for both HF and HFSC biology and local tissue homeostasis (Botchkarev et al., 1999; Liao & Nguyen, 2014; Shwartz et al., 2020). Further studies are needed to investigate whether and how the potential interaction of these genes with other genes or proteins, in conjunction with seasonal changes, contributes to the pathogenesis of CFA, particularly in the context of dermatomal neural modulation of the skin.

Several limitations of this study need to be considered. Firstly, for the low-pass WGS with imputation, we used a reference panel comprising 676 dogs covering 91 dog breeds, of which four were Rhodesian ridgebacks. This small number of Rhodesian ridgebacks may lead to inaccurate imputation affecting causal variant discovery. Future studies would benefit from high-coverage whole-genome sequencing data to validate imputed variants and enhance the accuracy and completeness of variant discovery. A second limitation of this study is the absence of functional validation of the imputed variants. Therefore, no direct conclusions can be drawn regarding their biological effects or causal involvement in the pathogenesis of CFA.

Based on the variable phenotypical expression in CFA-affected dogs, a more complex genetic basis is more likely. This assumption of a polygenic basis for CFA is supported by findings in a genomic study among atypical recurrent flank alopecia-affected (aRFA) Cesky Fousek dogs (Neradilova et al., 2022). The study indicated that aRFA is a skin disorder similar to CFA, with a complex inheritance pattern. Future studies are needed to validate the polygenic basis of CFA. Calculating the heritability may further confirm the mode of inheritance, but it could not be performed because of the lack of pedigree data for the control dogs.

Overall, the present study identifies a potential genomic region of interest and lays a foundation for future studies.

## Conclusion

This low-pass whole-genome sequencing-based study suggests that CFA in Rhodesian ridgebacks is a skin disorder that is unlikely to be caused by a single genetic defect. We identified a potential genomic region and several candidate genes with previously reported functional relevance to HF biology, suggesting that the disorder is genetically heterogeneous. However, given the modest sample size and the absence of functional validation, the putative associations should be interpreted cautiously and regarded as preliminary. Additional studies involving larger cohorts, refined mapping, and functional analyses are required to establish whether the highlighted genomic region and candidate genes contribute to the pathogenesis of CFA in Rhodesian ridgebacks. Our findings provide preliminary genomic insights and may help guide future studies aimed at clarifying the genetic and biological mechanisms underlying CFA, ultimately benefiting breeding strategies.

## Ethical approval

The authors declare that no ethical approval was required for this study. Before inclusion in the study, skin biopsies were taken by the attending practitioner for the clinical diagnosis of CFA, which did not require ethical approval. We obtained written consent from the participating owners to collect blood samples for DNA isolation and their secondary use for future scientific research. All data were pseudonymized to safeguard the owner’s privacy.

## Funding

The European Society of Veterinary Dermatology Practitioners Research Grant 2018.

## Ethical approval

The authors declare that no ethical approval was required for this study. Before inclusion in the study, skin biopsies were taken by the attending practitioner for the clinical diagnosis of CFA, which did not require ethical approval. We obtained written consent from the participating owners to collect blood samples for DNA isolation and their secondary use for future scientific research. All data were pseudonymized to safeguard the owner’s privacy.

## CRediT authorship contribution statement

**Millie U.M.Y. Verschuuren-Tjoeng:** Writing – original draft, Resources, Project administration, Methodology, Investigation, Funding acquisition, Conceptualization. **Lieke Vree:** Writing – review & editing, Formal analysis. **Claudia Rozendom:** Writing – review & editing, Investigation. **Yvette Schlotter:** Writing – review & editing. **Ronette Gehring:** Writing – review & editing, Supervision. **Frank G. van Steenbeek:** Writing – review & editing, Writing – original draft, Visualization, Validation, Supervision, Resources, Project administration, Methodology, Investigation, Formal analysis, Data curation, Conceptualization.

## Declaration of competing interest

The authors declare that they have no known competing financial interests or personal relationships that could have appeared to influence the work reported in this paper.

## Data Availability

The data presented in this study are deposited in the SRA repository (PRJNA1402291).

## Appendix Supplementary materials

Image, application 1

Image, application 2

Image, application 3

Image, application 4
