# Supplementary figures and images for: Genetic insights into canine flank alopecia in Rhodesian ridgebacks: identifying candidate genes
Source: Vet Anim Sci. 2026 Apr 21;33:100670. doi: 10.1016/j.vas.2026.100670 (PMC13186071; doi:10.1016/j.vas.2026.100670)

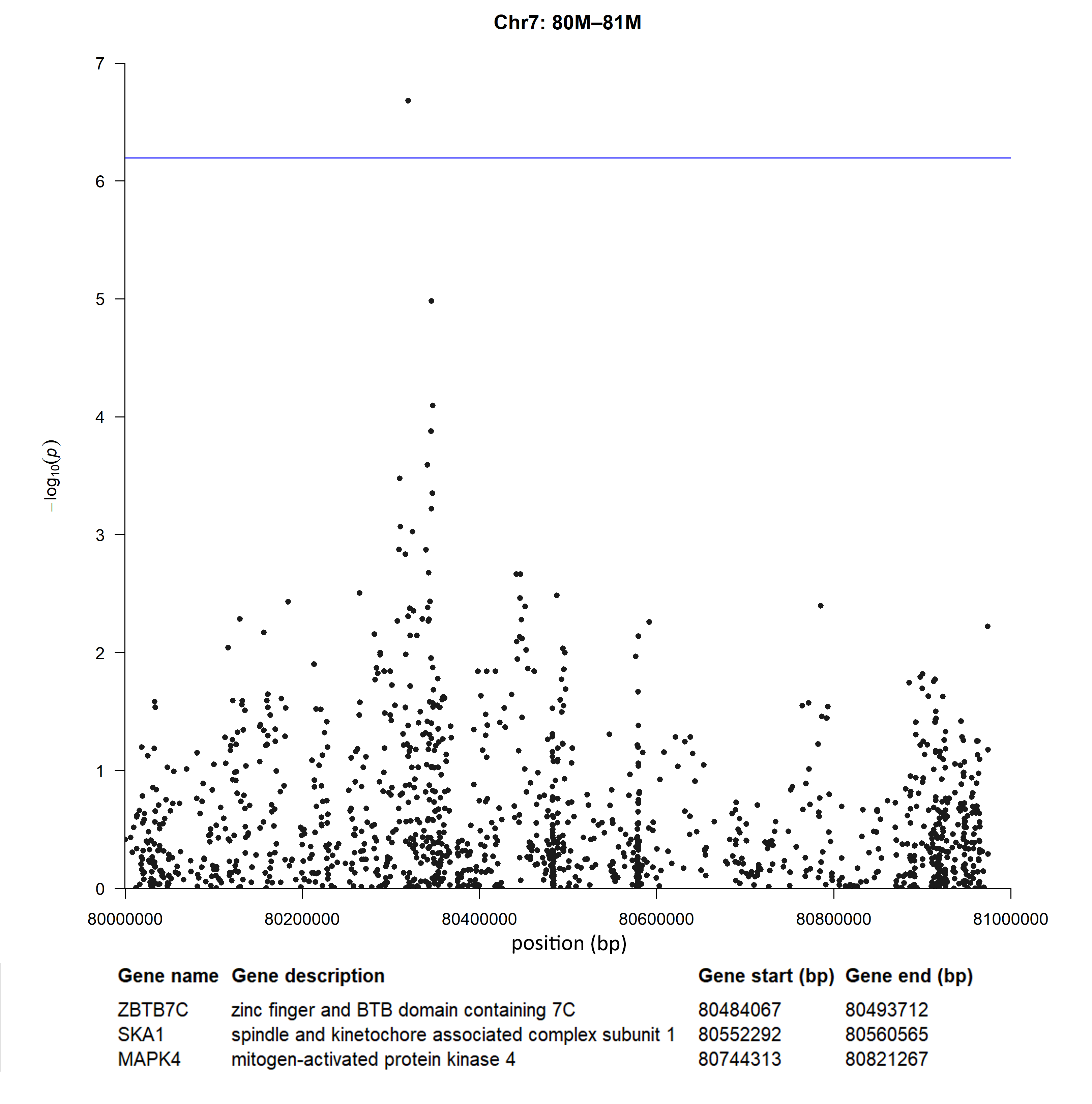

Supplement: Supplementary file 1 [file mmc1.zip › mmc1.tiff]
